# Evaluation of the clinical effects of non‐invasive prenatal screening for diseases associated with aneuploidy and copy number variation

**DOI:** 10.1002/mgg3.2200

**Published:** 2023-06-24

**Authors:** Shaohua Zhu, Chunyang Jia, Shengju Hao, Qinghua Zhang, Jing He, Xing Wang, Pengwu Lin, Yuanyuan Guo, Yigang Li, Xuan Feng

**Affiliations:** ^1^ Medical Genetic Centre, Gansu Maternity and Child‐Care Hospital Lanzhou China; ^2^ Gansu Provincial Clinical Research Center for Birth Defects and Rare Diseases Lanzhou China

**Keywords:** copy‐number variation (CNV), microdeletion/microduplication syndromes (MMS), non invasive prenatal screening Plus (NIPS‐Plus), positive predictive value (PPV)

## Abstract

**Background:**

To explore and compare the clinical effects of high‐resolution non‐invasive prenatal screening (NIPS‐Plus) for common/uncommon chromosomal aneuploidy and microdeletion/microduplication syndromes (MMS).

**Methods:**

The current prospective study included a total of 25,380 pregnant women who performed NIPS‐Plus, and amniocentesis was performed on women with MMS with the screening results to diagnose patients with suspected MMS.

**Results:**

There were 415 samples with positive results for NIPS‐Plus, included 275 with aneuploidy and 140 with MMS. After diagnosis by amniocentesis, 188 cases were confirmed as true positive, included46 cases of T21, 9 cases of T18, 1 case of T13, 34 cases of SCA, 41 cases of other chromosomal euploidy and 57 cases of MMS. In addition, no false negative cases were found, MMS was classified with 5 Mb with the cutoff value, and the PPV of different fragment size was counted, respectively.

**Conclusion:**

We found that the corresponding PPV was 44.66% with the fragment of copy number variation (CNV) being less than or equal to 5 Mb, and when it was greater than 5 Mb, the PPV was 29.73%, which suggested that NIPS‐Plus was more suitable for screening the PPV of small fragment abnormalities. NIPS‐Plus has a good application effect in routine aneuploidy screening and had the best detection effect for T21; moreover, it performed well in screening of MMS and had better detection effect on MMS with CNV fragment length less than 5 Mb. Based on the current results, we suggested that NIPS‐Plus should be used as a comprehensive elementary prenatal screening method for all pregnant women, but for MMS caused by abnormal large fragment CNV, the detection method and efficiency still need to be improved.

## Graphical abstract

In this work, we collected prenatal NIPS‐PLUS information from 25,380 pregnant women, and based on this information, we further analyzed patients with syndromes with different CNV fragment sizes. Based on the current findings, we recommend that NIPS‐Plus be used as a comprehensive primary prenatal screening method for all pregnant women, but for MMS caused by abnormally large fragments of CNV, the detection method and efficiency need to be improved. This is the first large‐scale statistical study based on NIPS‐PLUS results on pregnant women in northwest China, which has guiding significance for local prenatal screening.

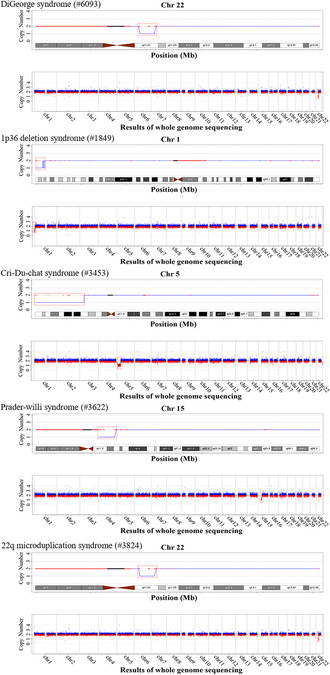

## 1 INTRODUCTION

Chromosomal abnormalities (aneuploidy and segment loss & gain) are common in humans, which are generally caused by non‐segregation of chromosomes at the meiotic stage of embryos before implantation, erroneous separation or mitotic replication errors (Vanneste et al., 2009). In most cases, these errors could lead to stagnant embryo growth, failure of implantation, or spontaneous termination in the early stages of pregnancy, which could lead to miscarriage (Garcia‐Herrero et al., 2016); moreover, around 1%–1.7% of fetuses with chromosomal abnormalities still have normal development ability even if they are affected by chromosomal abnormalities (Wapner et al., 2012). If the prenatal diagnosis is not detected, it may continue into the second and third trimesters of pregnancy until it shows chromosomal disease syndrome at their birth (Galindo et al., 2009).

Invasive amniocentesis or chorionic villus sampling to obtain prenatal specimens for karyotyping and Copy Number Variation Sequence (CNV‐seq) has become an important diagnostic method for detecting chromosomal abnormalities, but first trimester screening (FTS) through these methods may increase the indication of increased risk of abnormal fetal development (include biochemical and ultrasound screening) (Levy & Wapner, 2018). In addition, the standards for villus cell sampling and culture are quite strict, and the success rate of culture and the resolution of the results are very low without professional training and long practice. Therefore, this method was difficult to achieve satisfactory results, generally (Teles et al., 2017).

With the discovery of cell‐free fetal DNA (cffDNA) and the development of technology of next‐generation sequencing (NGS), revolutionary changes have taken place in reproductive medicine and medical genetics (Chitty & Lo, 2015). Non‐invasive prenatal screening (NIPS) is a new screening method that adopts cffDNA to detect fetal chromosomal abnormalities based on NGS. This method can effectively identify chromosomal abnormalities, such as major aneuploidy and rare autosomal aneuploidy (Scott et al., 2018). Previous studies have shown that deeper sequencing and more diversified analysis could simultaneously screen not only for common aneuploidies and but also for MMS (Shaffer & Norton, 2018). With the widespread adoption of NIPS in the past 9 years, more pregnant women who have been prompted by traditional screening methods to be at risk (high‐risk or low‐risk) pregnancy choose NIPS‐Plus for further testing (Liang et al., 2019). The test results show that the sensitivity and specificity of NIPS for common chromosome aneuploidy (trisomy) and sex chromosome aneuploidy (SCA)tests (Mazloom et al., 2013; Samango‐Sprouse et al., 2013) were significantly higher than those of traditional maternal serological screening (Bianchi et al., 2014; Song et al., 2013). In addition, this change in prenatal screening methods has reduced invasive test requests by 40% (Wong & Lo, 2016).

Nowadays, NIPS is still improving: in addition to screening for common aneuploidies, the technology is now available for microdeletion/microduplication syndrome (MMS) caused by segmental chromosomal abnormalities (Benn & Grati, 2018). MMS was also similar to aneuploidy and could be associated with many serious chromosomal disorders (Watson et al., 2014), and for instance, the micro‐deletion of the 22q11.21 region of the chromosome is associated with DiGeorge Syndrome (DGS) and it is also one of the most frequently occurring MMS. Neonatus suffered from hereditary diseases could bring some worries and subsequent effects on the children and their families. Several clinical studies have suggested that NIPS‐Plus could be adopted as a supporting detection method for MMS due to its higher genome coverage (Liu et al., 2016; Shi et al., 2021).

**FIGURE 1:**
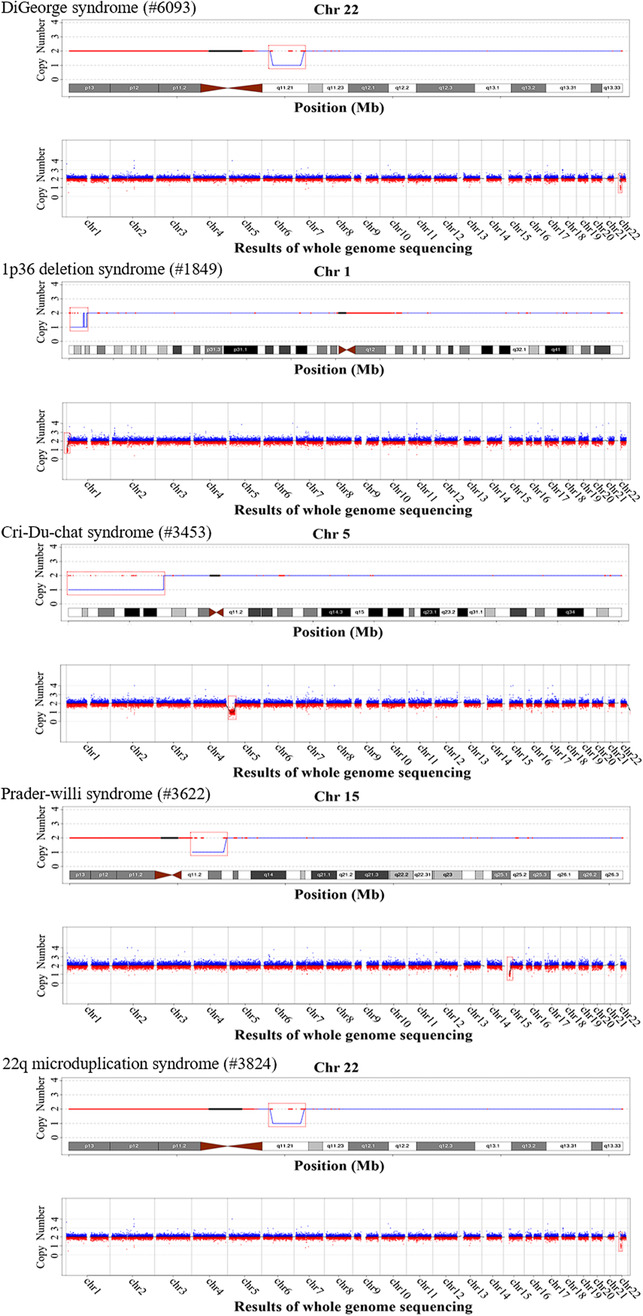
Confirmatory analysis of true‐positive microdeletion/microduplication syndrome (MMS) detected by NIPS‐Plus. Analysis results consist of segment‐specific CNV‐Seq analysis (top) and NIPS‐Plus detection (bottom); copy‐number variations (CNVs) are highlighted through red dotted boxes.

In most cases, CNVs exist in normal polymorphic forms, and only a few CNVs are associated with genetic diseases. Nevertheless, the microdeletion/duplication syndromes caused by Pathogenic CNV (pCNVs) still play an important role in human genetic diseases. For common chromosomal MMS (Sebat et al., 2004), such as Williams‐Beuren Syndrome (WBS, around 1.6 Mb heterozygous deletion at 7q11.23) and Angelman/Prader‐Willi syndrome (AS, PWS, 15q11‐13), DGS (22q11.2 loss of heterozygosity), 15q26 overgrowth syndrome (around 3.05 Mb gain at 15q26) are all caused by the microdeletion or duplication of CNVs or chromosome segments. In these chromosomal diseases, the location of CNVs are distributed in different regions of the genome, and the size of fragments are also different. Statistical parameters, such as detection rate, positive predictive value (PPV) and sensitivity, calculated based on a large number of NIPS cases are of great significance in prenatal screening. Therefore, the analysis and comparison of the above parameters has become one of the focuses in current study (Figure 1).

In current study, we conducted a prospective study of 25,830 pregnant women with no abnormalities in the first trimester through NIPS‐Plus, the mainstream technology in prenatal screening for screening common chromosome aneuploidy and MMS. Classify the results of NIPS‐Plus, the positive results will be combined with invasive prenatal confirmatory tests, such as karyotyping and CNV‐seq for diagnosis, so as to calculate the detection rate, true or false positive rate, sensitivity, specificity and other statistical parameters (Iafrate et al., 2004; Redon et al., 2006); moreover, based on statistical data, we tried to evaluate the impact of CNV fragment size on PPV. This has reference significance for patients to make informed decisions according to pregnancy management and reduce the incidence of children with severe MMS (Liang et al., 2019). Chromosome karyotype analysis of amniotic fluid of pregnant women, considering the low resolution of this method, it is difficult to obtain satisfactory results for some detections involving microduplication or deletion of fragments. Therefore, in addition to karyotype analysis, we also detect small fragment abnormalities of the genome through CNV‐seq, and the main purpose is to explore the application value of NGS in prenatal screening and diagnosis.

## 2 MATERIALS AND METHODS

### 2.1 Study oversight

The medical genetic center of this hospital is approved and authorized by the Provincial Health Commission, so it has qualified qualifications. All pregnant women undergoing testing were registered in the Medical Genetics Center in accordance with the standard procedures established of NIPS, and fill in the supporting informed consent procedure of NIPS‐Plus, included warnings and risks of restricted groups, methods of test, sample types and categories of screening genetic diseases. The consent form also included details of the screening supporting insurance plan, statements of relevant laws and national ethics guidelines. Each pregnant woman received laboratory application and pre‐test consultation information, which involved trisomy 13,18,21, sex chromosome aneuploidy, moreover, it also included positive predictive value (PPV), negative predictive value (NPV), sensitivity and specificity of CNV‐seq results. In particular, this study did not provide free prenatal examination to pregnant women in exchange for participating in the study, so the objectivity and reliability of the study were ensured as much as possible, and there was no adverse effect on the results of the study. Every inspector in the laboratory of the Medical Genetics Center has qualified professional qualifications, and ensures that they read and understand all the contents of the informed consent form. All pregnant women have purchased an insurance plan with standard NIPS‐Plus testing from insurance company (Pingan Insurance Company Ltd., Gansu Province, China).

### 2.2 Cases demographics

The 25,830 general population of pregnant women who requested screening for fetal chromosomal abnormalities were recruited from February 2017 to August 2021 to participate in the study. In accordance with the optimal recommendations of NIPS testing, the pregnant women received NIPS‐Plus at 14–20 weeks. Based on all samples collected, the median age of pregnant women was 35 weeks of age and most of them were younger than 35 years old, and only a few were in the high childbearing age, included 7483 (66.52%) pregnant women aged more than 35 years high‐risk pregnancies, 17,183 (28.97%) pregnant women aged less than 35 years low pregnancy at risk and 1167 (4.51%) pregnant women of undisclosed age. Blood samples are generally collected early in the second trimester. The gestational age of all samples was ensured to be within the standard range (range 11–39 weeks), which is the current standard practice in China. The ENET algorithm is used to calculate the fetal fraction (FF) for all pregnancies (Kim et al., 2015).

### 2.3 NIPS‐Plus

The standard for collecting peripheral blood samples is 10 mL, collected in Streck tubes (Streck, USA), stored at 4°C, and subjected to subsequent experiments within 96 h. Extract about 8 ng cffDNA from the plasma sample obtained by centrifugation, and the range of cffDNA concentration was between 0.05 and 0.7 ng/μL, which meets the experimental requirements. It should re‐extract the cffDNA when the concentration of the extracted cffDNA is not within this range. According to the latest experimental Standard Operation Procedure (SOP) provided from Berry genomics company (Berry Hekang Biotechnology Corporation Ltd., Beijing, China), the steps in the traditional NIPS process were optimized (Liang et al., 2013), and the cffDNA library was directly constructed without amplification. Based on NGS method, after quality control, the cffDNA library was sequenced on the Next Seq CN 500 platform (Berry Gene and Illumina), and the amount of data obtained was about 20 Mb, each READs was 45 bp, consisting of a 37 bp target sequence and 8 bp index (Shi et al., 2021). Prune the sequenced READs to generate 36 bp RAW DATA genome sequences (fastq). Adopted the RUPA algorithm developed based on Berry Genomics, RAW DATA was aligned in GRCh37, and finally obtained the uniquely mapped READS (10–19 M). These uniquely mapped reads are assembled into non‐overlapping and continuous BINs, and the unit of each BIN was 100 kb, and further filtered to remove BINS with low coverage and with abnormal GC content (less than 30% or more than 70%). The quality of the final sequencing data must conform to the following standards: Q30 > 95%, the redundancy rate after sequencing <2%, and the CV of chromosome ratio <1%.The ENET algorithm was adopted to calculate the fetal DNA ratio (FDR) of pregnancies (Kim et al., 2015), and for cases with FDR less than 4%, it would be notified to perform a second blood sampling, which ensures the effectiveness and reliability of NIPS‐Plus test results (Shi et al., 2021).

Chromosome aneuploidy is judged based on the chromosome *Z*‐score of its corresponding CNV interval. According to the standard of *Z*‐score, when it less than −3 indicates deletion (CN = 1), greater than 3 indicates duplication (CN = 3), and *Z*‐score between −3 and 3 indicates it is within the normal range, that isdimorphism (CN = 2). Pregnant woman continued to be pregnant until full term if the corresponding cases with negative results of NIPS‐Plus, while the suspected cases with positive results of NIPS‐Plus would be recommended to amniocentesis to investigated and verified suspected fetal chromosomal aneuploidy, identity information of the selected samples was removed before testing for CNV, and all the samples were randomly rearranged and blinded by laboratory and bioinformatics personnel. The results of test were compared with karyotype or microarray results to calculate sensitivity and specificity. Adopted the chromosome karyotype analysis (G‐banding) to diagnose T13, T18, T21 trisomy and sex chromosome aneuploidy (SCA), and CNV‐Seq was adopted to analyze and diagnose the suggestive of MMS abnormalities or another segmental aneuploidy (Liang et al., 2014; Wang et al., 2014; Zhou et al., 2021). The Results of CNV‐seq was located and screened based on public genetic databases including DECIPHER, Database of Genomic Variants (DGV), GenomAD and Online Mendelian Inheritance in Man (OMIM), the pathogenicity of CNV was assessed according to the latest guidelines for interpretation and reporting of postnatal constitutional copy number variants of American College of Medical Genetics and Genomics (ACMG) and guidelines for interpretation of sequence variations of ACMG (Richards et al., 2015; Riggs et al., 2020). Before the age of 1 year, the newborns had basic check‐ups at local hospitals every three months to check for any signs of suspected or potential clinical disease.

## 3 RESULTS

### 3.1 Fetuses with suspected chromosomal abnormalities

The study population consisted of 25,830 pregnant women, who were detected for clinically significant fetal aneuploidy and MMS through NIPS‐Plus. A total of 415 fetuses (1.61%) were suspected of pathogenic or likely pathogenic chromosomal abnormalities (Table 1). Among these 415 patients, 275 (66.27%) were positive for chromosomal aneuploidy, and the other 140 (33.73%) were tested positive for MMS, involving CNV of varying fragment sizes. Cases with suspected positive screening results were recommended for amniocentesis. Patients with aneuploidy conducted karyotype analysis, and patients with suspected positive MMS conducted CNV‐Seq analysis, take corresponding methods to diagnose positive and negative for these patients.

**TABLE 1:** Detection performance of NIPS‐Plus for chromosomal disorders and syndromes in 25,830 pregnancies.

Fetal aneuploidies	TP	FP/FPR	PPV (%)	TN	Sensitivity (%)	FN/FNR (%)	NPV (%)	Specificity (%)
*Typical trisomies*	56	31	64.37	25,273	100	0/0	100	99.87
T21	46	10	82.14	25,312	100	0/0	100	99.96
T18	9	11	45	25,364	100	0/0	100	99.96
T13	1	10	9.09	25,367	100	0/0	100	99.96
*Rare trisomies*	41	119	25.63	25,171	100	0/0	100	99.52
*SCAs*	34	90	27.42	25,706	100	0/0	100	99.65
47,XXX	7	3	70	25,370	100	0/0	100	99.98
47,XXY	14	0	100	25,366	100	0/0	100	100
47,XYY	4	0	100	25,376	100	0/0	100	100
45,XO	9	87	9.38	25,351	100	0/0	100	99.66
*MMS*	57	83	40.71	25,690	100	0/0	100	99.68
PWS	0	1	0	25,829	100	0/0	100	99.99
DGS	2	5	28.57	25,823	100	0/0	100	99.98
22q dup syndrome	1	2	33.33	25,827	100	0/0	100	99.99
1p36 del syndrome	1	10	9.09	25,819	100	0/0	100	99.96
<5 Mb	46	57	44.66	25,234	100	0/0	100	99.77
≥5 Mb	11	26	29.73	25,350	100	0/0	100	99.89

Abbreviations: DGS DiGeorge syndrome; FN/FNR, false negative and false negative rate; FP/FPR, false positive and false positive rate; MMS, microdeletion/microduplication syndromes; NPV negative predictive value; PPV positive predictive value; PWS Prader–Willi syndrome; SCA, sex chromosome aneuploidy; T13 trisomy 13; T18 trisomy 18; T21, trisomy 21; TN true negative; TP, true positive.

### 3.2 Fetuses with suspected trisomies and SCAs

Among the 415 positive NIPS‐Plus results, 87 cases suggested a high risk of T21, T18, or T13 (Table 1), and there were 56 cases of T21, 20 cases of T18, and 11 cases of T13. but there were 31 false‐positive (FP) test results, included 10 cases of T21 (FPS), 11 cases of T18 and 1 case of T13, and the corresponding positive predictive values (PPV) were 82.14% (T21), 45.00% (T18) and 9.09% (T13), respectively; moreover, after follow‐up medical inquiry for patients, no false negative (FN) cases were identified, which indicated that the negative predictive value (NPV) of the three aneuploidies is 100%. There were 160 cases with positive results through NIPS‐Plus detection of rare autosomal aneuploidy, after corresponding diagnosis, 119 cases showed false positive (FP) results, that is, the NIPS‐Plus results showed FP and the positive predictive value (PPV) of rare autosomal aneuploidy was 26.63%. A total of 124 cases were showed positive for SCA, included 96 cases (77.42%) of Turner syndrome, 10 cases (8.06%) of triple X syndrome (47,XXX), and 4 cases (3.23%) of Jacob syndrome (47, XYY), 14 cases (11.29%) of Kline‐felter syndrome (47, XYY). In the subgroup (96 cases) of suspected Turner syndrome, 87 cases were incorrectly identified as 45,XO, which is a FP result, and the corresponding PPV was 9.38%; in the subgroup (10 cases) of suspected triple X syndrome, 3 cases of were incorrectly identified as 47,XXX, and the corresponding PPV was 70%. The remaining two SCA subgroups (47, XXY and 47, XYY) were correctly identified and no FP results occurred, so the PPV was 100%.

According to the results of NIPS‐Plus for MMS, there were 140 cases of pathogenic or likely pathogenic CNV samples, and 11 cases of MMS were related to classic chromosomal diseases, included1 suspected case of (Prader‐Willi Syndrome) PWS, 7 suspected cases of (DiGeorge Syndrome) DGS syndrome, and 3 suspected cases of 22q11.2 microduplication syndrome. The diagnostic results of amniocentesis and CNV‐seq suggested that the PPV of these classic MMS were all less than 50%, 1 case of suspected PWS the above‐mentioned was FP, and the corresponding PPV was 0%, 7 cases of suspected DGS included 2 TP and 5 FP, and the corresponding PPV was 28.57%, 3 cases of suspected 22q11.2 microduplication syndrome included 1 TP and 2 of FP, and the corresponding PPV was 33.33%, 11 cases of suspected 1p36 microdeletion syndrome included 1 TP and 10 of FP, and the corresponding PPV was 9.09%. In addition to the 11 typical MMS cases described above, there were 129 cases in which the screening results showed other CNVs. The names of these specific MMS could not be recognized in any current database, so these of CNVs were classified as MMS of non‐classical syndromes. In addition, the results of the study showed that for CNVs with different fragment length ranges, the corresponding PPVs were also different. Taking 5 Mb as the cutoffvalue, there were 37 cases with CNV fragment lengths greater than or equal to 5 Mb, included 11 cases of TP and 26 cases of FP, the corresponding PPV is 29.73%; and 103 samples with CNV fragment length less than 5 Mb, including 46 cases of TP and 57 cases of FP, the corresponding PPV is 44.66%. All the statistical results of this study, whether pathogenic or non‐pathogenic CNV, are analyzed by the same algorithm. Therefore, they can be presented as universal utility evidence in the genome, so as to reflect the integrity and reliability of the current study.

It should be noted that among all the NIPS‐Plus cases involved in current study, none of the pathogenic chromosomal abnormalities was missed. So, both the negative predictive value (NPV) and sensitivity in current study were 100%. Moreover, for these of cases, we conducted random follow‐up medical inquiry for patients to newborns within 3–6 months after birth, there were no FN reports about chromosomal aneuploidy and SCA were found, and the follow‐up medical inquiry is still continuing.Our results effectively proved that continuity and integrity of current study,and also suggested that although the NPV of current study was ideal, it did not exclude the possibility of encountering FN cases in the future, because various previous studies have shown that the mosaic phenomenon of placenta and fetus was one of the reasons that may lead to false negative (Liang et al., 2019).

## 4 DISCUSSION

NIPS technology has made considerable progress and has become a routine and reliable method in clinical prenatal screening and was widely accepted by pregnant women.Although NIPSwas suitable for detecting common fetal triploidy and SCA abnormalities, it was true that the clinical effect of detecting rare chromosomal diseases and syndromes caused by MMS was still controversial (Evans et al., 2016; Rose et al., 2016). The positive aspect was that no matter what method is adopted, the purpose of prenatal screening was to prevent the newborns with severe chromosomal disease or syndrome, even if NIPS has only moderate or low PPV in screening for some chromosomal disease, it still has sufficient sensitivity to identify most fetuses with rare chromosomal disease or syndrome; and it should be noted that the patients with rare chromosomal disease or MMS could not be detected by conventional ultrasound scans; the negative aspect wasthe concerns about the low PPV of NIPS‐Plus results for chromosomal aneuploidy and the many uncertainties associated with MMS because the results with likely pathogenic (LP) or variant of uncertain significance (VUS) could cause great difficulties to genetic counselingeven increase unnecessary invasive surgery and transferred the pressure to couples. The current NIPS guidelines also clearly pointed out that ACMG advocates the adoption of NIPS for the detection of common aneuploidies, but it alsosuggested that the detection procedure should be combined with the invasive test results of positive results (Gregg et al., 2016; Liang et al., 2019).

The current study showed the results and advantages of NIPS‐Plus in detecting pathogenic chromosome aneuploidy and MMS by detecting NIPS‐Plus in 25,830 pregnant women. According to statistical data, NIPS‐Plus has different applicability in detecting typical aneuploidy,for T21, which appeared most frequently in the study (total positive cases were 56), the PPV was very high, reaching 82.14%. For T18, the PPV was medium, reaching 45.00%, while for T13, the PPV was very low, only 9.11%. Our results had similar trend to previous studies, Shi et al. conducted a statistical study of NIPS‐Plus based on 36,970 pregnant women,their results showed that the PPV of T21, T18 and T13 reached 81.3%, 47.6% and 17.6%, respectively (Shi et al., 2021). Compared with our statistical results, the PPV of T21 is also the highest, and T13 is the lowest; it is not surprising, because the sample size we used is also similar to Shi et al. It is worth noting that we have achieved reliable results, but itdid not prove that our results were uncontroversial.Compared with another study, our results also have some differences, Liang et al. conducted a NIPS‐Plus screening study on 94,085 pregnant women, and conducted statistical studies on PPV, specificity, and sensitivity (Liang et al., 2019). According to their results, the overall PPV of T21 and T18 was very high, reached 95% and 82%, respectively,and the PPV of T13 is at a moderate level, reached 46%. The statistical results of the current study were consistent with the results of Liang et al. from trend comparison: the PPV of T21 is the highest and T13 is the lowest. However, if we only discuss the specific value of PPV, there was still a great disparity between the current study and the results of Liang et al. In any case, the large‐scale NIPS‐Plus was closely related to population distribution and size of cases. The current study had similar trends and even similar results to the previous studies mentioned above, which could also prove that the it had certain guiding significance for the subsequent development of larger‐scale testing.

A total of 140 cases of pathogenic CNV (pCNV) were detected in current study, according to the fragment size of pCNV, we divided them and adopt 5 Mb as a cutoff value to classify the pCNVs detected in the effective reading array. Among them, there were 103 cases of pCNV less than 5 Mb, and 37 cases of pCNV greater than 5 Mb. We calculated PPV of the two groups of pCNVs according to the fragment size, the results of statistical showed that for pCNVs less than 5 MB, the PPV was at the medium level, reaching 44.66%; and for pCNVs greater than 5 Mb, the PPV level was just at low level, only 29.73%. The results suggested that NIPS‐Plus has different PPVs for CNVs with different fragment sizes, and compared with large fragments of pCNVs, the small fragments of pCNVshad higher PPV,this may be related to the low sequencing depth of NIPS‐Plus or difference of the dataset, but statistical dataindicated that NIPS‐Plus was more suitable for pCNVs with smaller fragments. Previous studies have analogous reports: in a study about NIPS‐Plus, Pei et al. classified pCNVs with different fragment sizes, and performed PPV with 10 Mb as a cutoff value, their results showed that: PPV of CNV that fragment length less than 10 Mb was significantly higher than that of greater than 10 Mb. In addition, Hu et al. conducted a statistical study about NIPS‐Plus on 8141 cases (Hu et al., 2019), their results showed that the PPV of NIPS‐Plus was higher when the fragment length of CNV was smaller. The above‐mentioned studies could show that NIPS‐Plus was more suitable for CNV with smaller fragment length.

According to the data statistics of PPV of typical MMS, 22 suspected MMS cases were detected (Table 1), the PPV of DGS and 22q11.2 micro‐duplication syndrome were 28.57% and 33.33% respectively, with medium level, and the PPV of 1p36 micro‐deletion syndrome was only 9.09%, with low level, moreover, only 1 FP case of PWS was detected. In the study of Liang et al., a total of 32 positive typical MMS cases were detected through NIPS‐Plus, there were 23 TP cases, and DGS had the largest number of TP cases, with a total of 13 cases, and corresponding PPV was also the highest with 92.9%, for 22q11.2 micro‐duplication syndrome, there were 4 cases and the corresponding PPV reaches 66.7%, and 1p36 micro‐deletion syndrome was the lowest (0%). The statistical results of PPV incurrent study were lower than the above results, but the trends wereconsistent, it would indicate that our statistical results were reasonable with sufficient sensitivity and specificity, which could be adopted to screen and detect potential CNVs from the whole genome of the fetus.

The comparison results with previous studies not only suggested that our results were reliable but also showed that some differences in the results of case statistics in different scale of population. However, according to the current study, the PPV of SCA and MMS detected through NIPS‐Plus was very low, which still fails to achieve the satisfactory results, it would cause anxiety for pregnant women and additional unnecessary clinical trials (Liang et al., 2019). Previous studies have shown that the PPV of NIPS‐Plus could be improved through deeper sequencing, Martin and his colleagues analyzed PPV of NIPS under different depths of sequencing in. A subgroup for comparison was included in their study population, the subgroup adopted deeper sequencing to conduct the NIPS‐Plus detection and found that the PPV of MMS in this subgroup increased, the most obvious of which was DGS syndrome, that increased from 15.7% to 44.2% (Martin et al., 2018). In addition, increasing sequencing depth also has an important impact on PPV estimation of common trisomic abnormalities, and according to Bayindir et al., the increased sequencing depth was beneficial in reducing the false positive rate of common trisomies (Bayindir et al., 2015). Higher depth of sequencing would indeed lead to better results, but it also means that more costs need to be invested (Pös et al., 2019). The method of improving PPV of MMS by increasing depth of sequencing may not be really applied in clinical practice, because the charging price of NIPS‐Plus was also a factor that must be considered for the majority of patients, according to the current cost of NGS, NIPS based on low‐depth sequencing was still suitable for typical and other chromosomal abnormalities, Included MMS caused by pCNV (Chen et al., 2019; Deng et al., 2019).

## 5 CONCLUSIONS

The detection of chromosomal abnormalities and SCA through NIPS‐Plus had become more and more routine. Although the PPV of some MMS still need to be improved, it had a high‐quality effect in the detection of T21 and SCA. In addition, a number of study results, included ours, suggested that this technique showed high performance in detecting small fragments of pCNV. Although the PPV in typical MMS, such as DGS and 22q dup syndrome, achieved only a moderate or low levels, it was likely to be related to the depth of sequencing. With the reduction of sequencing cost, the depth of sequencing could continue to increase, which will improve the PPV and detection efficiency of MMS. Of course, we will also strive to improve the depth of sequencing and expand the number of cases and conduct a more comprehensive and in‐depth discussion in the follow‐up study, but now, it is still in the stage of trial and exploration.In terms of current application status, NIPS‐Plus will still adopt the low‐depth sequencing for detection, but with the popularization of sequencing technology, we can try to find the appropriate balance between detection efficiency and cost.

### AUTHOR CONTRIBTUIONS

*Conceptualization*: Shaohua Zhu and Qinghua Zhang. *Data curation*: Shaohua Zhu and Chunyang Jia. *Formal analysis*: Shaohua Zhu and Jing He. *Funding acquisition*: Xuan Feng. *Investigation*: Xing Wang and Pengwu Lin. *Methodology*: Shengju Hao. *Project administration*: Shengju Hao and Xuan Feng. *Resources*: Yuanyuan Guo and Chunyang Jia. *Software*: Shaohua Zhu and Yigang Li. *Validation*: Xing Wang. *Visualization*: Jing He. *Writing – original draft*: Shaohua Zhu and Chunyang Jia: *Writing – review & editing*: Shengju Hao and Xuan Feng.

## FUNDING INFORMATION

This research, including experimental design, sample collection, data analysis, and manuscript writing, was funded by the Gansu Provincial Department of Science and Technology Innovation Base and Talent Plan (21JR7RA680), the Major project of Gansu Maternal and Child Health Hospital (GSFY‐2021), and Clinical application of non‐invasive prenatal genetic testing technology in chromosomal microdeletion and microduplication syndrome (2017‐04‐50).

## CONFLICT OF INTEREST STATEMENT

The authors declare no conflict of interest.

## ETHICS STATEMENT

All the case recruitment procedures involved in current study were in full compliance with national ethical standards, and the study was approved by the Institutional Review Boards of Gansu Provincial Maternity and Child‐care Hospital (License number: 2021GSFY Ethical Review No. 65).

## Supporting information


Supplementary Files 1.

## Data Availability

Data available on request due to privacy/ethical restrictions.
